# Effects of the physiological parameters on the signal-to-noise ratio of single myoelectric channel

**DOI:** 10.1186/1743-0003-4-29

**Published:** 2007-08-08

**Authors:** Heather T Ma, YT Zhang

**Affiliations:** 1 Jockey Club Centre for Osteoporosis Care and Control, School of Public Health, The Chinese University of Hong Kong, Shatin, NT, Hong Kong, China; 2 Joint Research Centre for Biomedical Engineering, Department of Electronic Engineering, The Chinese University of Hong Kong, Shatin, NT, Hong Kong, China

## Abstract

**Background:**

An important measure of the performance of a myoelectric (ME) control system for powered artificial limbs is the signal-to-noise ratio (SNR) at the output of ME channel. However, few studies illustrated the neuron-muscular interactive effects on the SNR at ME control channel output. In order to obtain a comprehensive understanding on the relationship between the physiology of individual motor unit and the ME control performance, this study investigates the effects of physiological factors on the SNR of single ME channel by an analytical and simulation approach, where the SNR is defined as the ratio of the mean squared value estimation at the channel output and the variance of the estimation.

**Methods:**

Mathematical models are formulated based on three fundamental elements: a motoneuron firing mechanism, motor unit action potential (MUAP) module, and signal processor. Myoelectric signals of a motor unit are synthesized with different physiological parameters, and the corresponding SNR of single ME channel is numerically calculated. Effects of physiological multi factors on the SNR are investigated, including properties of the motoneuron, MUAP waveform, recruitment order, and firing pattern, etc.

**Results:**

The results of the mathematical model, supported by simulation, indicate that the SNR of a single ME channel is associated with the voluntary contraction level. We showed that a model-based approach can provide insight into the key factors and bioprocess in ME control. The results of this modelling work can be potentially used in the improvement of ME control performance and for the training of amputees with powered prostheses.

**Conclusion:**

The SNR of single ME channel is a force, neuronal and muscular property dependent parameter. The theoretical model provides possible guidance to enhance the SNR of ME channel by controlling physiological variables or conscious contraction level.

## Background

### Introduction

The surface myoelectric (ME) signal is an effective and important indicator of neuromuscular characteristics and inherent mechanisms underlying muscle activity. This accessible signal has been widely studied for diverse purposes, such as fundamental understanding of neuromuscular processes, diagnosis and therapy of neuromuscular diseases. Especially for amputee, features extracted from ME signals are adopted as parameters to control the powered prostheses, which is termed, ME control.

Proper measurement of ME control performance is crucial in determining feasible techniques for successful training for neuromuscular rehabilitation or multifunctional prostheses. Because the surface recorded ME signal is amplitude modulated corresponding to muscle contraction level, its amplitude is usually assumed as constant for nonfatiguing, constant-force and -angle contractions. However, estimate of ME signal amplitude is not constant due to its stochastic property. Variations around the mean value of the amplitude estimate are considered to be noise. It should be noticed that the "noise" used in this context is distinct from the interference residing in the ME signal measurement, such as the interferences arose from the recording electrodes and power line. In such a circumstance, signal-to-noise ratio (SNR), defined as the ratio of the amplitude of a desired signal to the amplitude of noise, can be used as a measure of the quality of an ME signal processor. Root-mean-square, mean-absolute-value (MAV), and mean-square-value (MSV) are generally used functions for the ME signal processor.

### Relevant research

Most of the research on factors that influence the SNR in the ME control has focused on signal processors, such as the effects of the averaging filter [1,2] and the nonlinearity of the processor [3,4]. In recent studies, Zhang et al. [5] employed the SNR to study the MSV processor based on the linear model, where the ME signal is modelled as a temporal and spatial summation of motor unit action potentials. The results of their study showed that the SNR nonlinearly increased with the increment of the contraction level, and its theoretic asymptote was equal to that which would result if the ME signal were modelled as a Gaussian random process. Clancy and Hogan [6] used the SNR as the standard metric to compare the performance of ME signal processors, MAV and RMS. They found that if the electromyographic density is Laplacian, the MAV processing is optimal in terms of SNR. Due to the different SNR computation, it is difficult to directly compare the results from Clancy and Hogan with those from Zhang's study. However, the theoretical results of both groups could be repeated in experiments, validating the respective modelling methods.

By the linear model, an ME signal is the temporal and spatial summation of the signals generated by all activated motor units. One merit of this model is that it lends itself to study individual ME channels and their interrelationship. Based on such a modelling scheme, Zhang et al. [5] indicated that the SNR, defined as the ratio of the MSV estimation at the channel output and the variance of the estimation, is largely influenced by the statistics of ME signals [7], which are determined by the neuromuscular physiology. However, only a few studies have reported on the effects of the interaction between the neuron and muscle on the SNR at the ME control channel output. The purpose of this paper is to investigate the effects of neuromuscular physiology on the SNR at the single ME channel output, to obtain a better understanding of the relationship between muscle contraction and ME control performance. If there is no special description, the SNR in this study refers to the ratio of the MSV estimation at the channel output and the variance of the estimation, the same in Zhang's research. A theoretical model will be proposed and simulations will be performed accordingly.

## Methods

### Model of Myoelectric (ME) Channel

**Figure 1:**
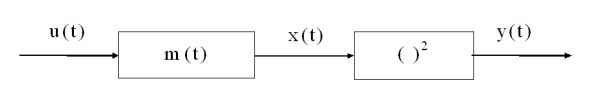
ME channel model for single motor unit. u(t) is the innervation process from MN, m(t) is the impulse response function of motor unit, and ( )^2 ^is the nonlinear processor with square operator [7].

An ME channel is the ME signal generation process of a single motor unit combined with a signal processor with a nonlinear function. Figure 1 shows a linear model of a single ME channel that was commonly used in previous studies [5,8].

A squarer is employed as the nonlinear processor, and the channel output is the convolution of the motor unit action potential (MUAP), m(t), with an innervation process u(t), which is the output of the motoneuron (MN). This model assumes that [8]: 1) u(t) is a stationary process with a mean firing rate *r*; 2) the inter spike intervals (ISIs) of a given MUAP train are statistically independent and thus u(t) is a renewal point process; 3) the motor unit process *x*(*t*) is assumed to have a mean of zero and is uncorrelated; and 4) muscle fatigue is negligible. To evaluate ME control performance, SNR was defined as the ratio of the MSV estimation at the channel output and the variance of the estimation. The definition of SNR in this study is the same to that in Zhang's investigation [5], as shown in Eq.1.

SNR=E2{y(t)}Var{y(t)}=rk−r,
 MathType@MTEF@5@5@+=feaafiart1ev1aaatCvAUfKttLearuWrP9MDH5MBPbIqV92AaeXatLxBI9gBaebbnrfifHhDYfgasaacH8akY=wiFfYdH8Gipec8Eeeu0xXdbba9frFj0=OqFfea0dXdd9vqai=hGuQ8kuc9pgc9s8qqaq=dirpe0xb9q8qiLsFr0=vr0=vr0dc8meaabaqaciaacaGaaeqabaqabeGadaaakeaacqWGtbWucqWGobGtcqWGsbGucqGH9aqpdaWcaaqaaiabdweafnaaCaaaleqabaGaeGOmaidaaOWaaiWaaeaacqWG5bqEcqGGOaakcqWG0baDcqGGPaqkaiaawUhacaGL9baaaeaacqWGwbGvcqWGHbqycqWGYbGCdaGadaqaaiabdMha5jabcIcaOiabdsha0jabcMcaPaGaay5Eaiaaw2haaaaacqGH9aqpdaWcaaqaaiabdkhaYbqaaiabdUgaRjabgkHiTiabdkhaYbaacqGGSaalaaa@4C26@

where m(t) represents the MUAP which is a function of time index t; y(t) is the ME channel output, i.e. the single motor unit output passed through the nonlinear processor; E(·) and Var(·) denote operations for calculating the expectation and variance calculation in time domain; k > r and

k=∫−∞∞m4(t)dt(∫−∞∞m2(t)dt)2.
 MathType@MTEF@5@5@+=feaafiart1ev1aaatCvAUfKttLearuWrP9MDH5MBPbIqV92AaeXatLxBI9gBaebbnrfifHhDYfgasaacH8akY=wiFfYdH8Gipec8Eeeu0xXdbba9frFj0=OqFfea0dXdd9vqai=hGuQ8kuc9pgc9s8qqaq=dirpe0xb9q8qiLsFr0=vr0=vr0dc8meaabaqaciaacaGaaeqabaqabeGadaaakeaacqWGRbWAcqGH9aqpdaWcaaqaamaapedabaGaemyBa02aaWbaaSqabeaacqaI0aanaaGccqGGOaakcqWG0baDcqGGPaqkcqWGKbazcqWG0baDaSqaaiabgkHiTiabg6HiLcqaaiabg6HiLcqdcqGHRiI8aaGcbaWaaeWaaeaadaWdXaqaaiabd2gaTnaaCaaaleqabaGaeGOmaidaaOGaeiikaGIaemiDaqNaeiykaKIaemizaqMaemiDaqhaleaacqGHsislcqGHEisPaeaacqGHEisPa0Gaey4kIipaaOGaayjkaiaawMcaamaaCaaaleqabaGaeGOmaidaaaaakiabc6caUaaa@4FA1@

**Figure 2:**
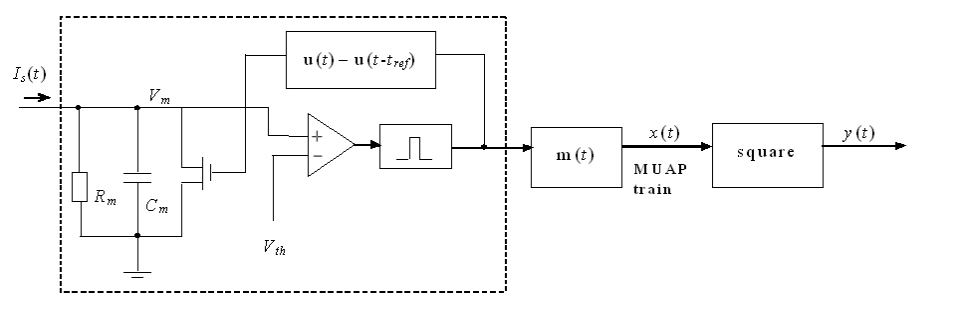
A model of ME channel including the MN firing mechanism, which is illustrated in the dashed line.

Equation 1 shows that the firing rate is a key factor that affects the SNR. Physiologically, the firing occurrence between the MN and muscle motor unit has a one-to-one relationship so that the firing rate only depends on the MN status. By introducing an integrate-and-fire (IF) mechanism to model MN the firing characteristics, the single ME channel can be modified as shown in Fig. 2. The modified model is based on three fundamental elements: an IF MN, a MUAP module, and a signal processor with a squarer function. The IF model is a simple but quite powerful model to describe a spiking cell. It includes two key aspects of neuronal excitability: a passive, integrating subthreshold phase and the generation of stereotypical impulses once a threshold is exceeded. The absolute refractory period (ARP) is modelled as a non-response time and realized by a switch controlled by a square pulse. The I_s_(t) is the gross stimulating current from the central nervous system (CNS), R_m _and C_m _are lumped membrane resistance and capacitance, respectively, and V_th _is the threshold for firing.

Physiologically, I_s_(t) is an excitatory drive function representing either the synaptic input or current elicited by an electrode. Investigators have asserted that the synaptic current input for a MN can be quantitatively measured as an injected constant current, which is termed the effective current [9-11]. This marks an important advance in the attempt to assess the operation of neuronal activity by introducing a much simplified input function instead of a complex mechanism regulating current delivery from the dendrite to the soma of the MN. As a result, a constant current stimulation was adopted in the model. Accordingly, the subthrehsold time course of the membrane potential was governed by the first-order differential equation:

CmdVm(t)dt+Vm(t)Rm=Is(t).
 MathType@MTEF@5@5@+=feaafiart1ev1aaatCvAUfKttLearuWrP9MDH5MBPbIqV92AaeXatLxBI9gBaebbnrfifHhDYfgasaacH8akY=wiFfYdH8Gipec8Eeeu0xXdbba9frFj0=OqFfea0dXdd9vqai=hGuQ8kuc9pgc9s8qqaq=dirpe0xb9q8qiLsFr0=vr0=vr0dc8meaabaqaciaacaGaaeqabaqabeGadaaakeaacqWGdbWqdaWgaaWcbaGaemyBa0gabeaakmaalaaabaGaemizaqMaemOvay1aaSbaaSqaaiabd2gaTbqabaGccqGGOaakcqWG0baDcqGGPaqkaeaacqWGKbazcqWG0baDaaGaey4kaSYaaSaaaeaacqWGwbGvdaWgaaWcbaGaemyBa0gabeaakiabcIcaOiabdsha0jabcMcaPaqaaiabdkfasnaaBaaaleaacqWGTbqBaeqaaaaakiabg2da9iabdMeajnaaBaaaleaacqWGZbWCaeqaaOGaeiikaGIaemiDaqNaeiykaKIaeiOla4caaa@4ADE@

Together with an initial condition, Eq.2 specifies the voltage trajectory of the subthreshold membrane potential. When the effective synaptic current of I_s_(t) is a step of constant current I_0 _switched on at t = 0, *V*_*m *_can be obtained by solving Eq.2 as,

Vm(t)=I0Rm(1−e−t/τm)+Vre−t/τm.
 MathType@MTEF@5@5@+=feaafiart1ev1aaatCvAUfKttLearuWrP9MDH5MBPbIqV92AaeXatLxBI9gBaebbnrfifHhDYfgasaacH8akY=wiFfYdH8Gipec8Eeeu0xXdbba9frFj0=OqFfea0dXdd9vqai=hGuQ8kuc9pgc9s8qqaq=dirpe0xb9q8qiLsFr0=vr0=vr0dc8meaabaqaciaacaGaaeqabaqabeGadaaakeaacqWGwbGvdaWgaaWcbaGaemyBa0gabeaakiabcIcaOiabdsha0jabcMcaPiabg2da9iabdMeajnaaBaaaleaacqaIWaamaeqaaOGaemOuai1aaSbaaSqaaiabd2gaTbqabaGcdaqadaqaaiabigdaXiabgkHiTiabdwgaLnaaCaaaleqabaGaeyOeI0IaemiDaqNaei4la8ccciGae8hXdq3aaSbaaWqaaiabd2gaTbqabaaaaaGccaGLOaGaayzkaaGaey4kaSIaemOvay1aaSbaaSqaaiabdkhaYbqabaGccqWGLbqzdaahaaWcbeqaaiabgkHiTiabdsha0jabc+caViab=r8a0naaBaaameaacqWGTbqBaeqaaaaakiabc6caUaaa@50F4@

where *τ*_*m *_is the membrane time constant and equals to *C*_*m*_*R*_*m*_, *V*_*r *_refers to the resting potential before stimulating which is set to zero. Obviously, the minimal sustained current to trigger an action potential, the threshold current, is *I*_*th *_= *V*_*th*_/*R*_*m*_. For any current *I*_0 _larger than *I*_*th*_, an output impulse will be generated at time *T*_*th*_,

Tth=τmln⁡(I0RmI0Rm−Vth).
 MathType@MTEF@5@5@+=feaafiart1ev1aaatCvAUfKttLearuWrP9MDH5MBPbIqV92AaeXatLxBI9gBaebbnrfifHhDYfgasaacH8akY=wiFfYdH8Gipec8Eeeu0xXdbba9frFj0=OqFfea0dXdd9vqai=hGuQ8kuc9pgc9s8qqaq=dirpe0xb9q8qiLsFr0=vr0=vr0dc8meaabaqaciaacaGaaeqabaqabeGadaaakeaacqWGubavdaWgaaWcbaGaemiDaqNaemiAaGgabeaakiabg2da9GGaciab=r8a0naaBaaaleaacqWGTbqBaeqaaOGagiiBaWMaeiOBa42aaeWaaeaadaWcaaqaaiabdMeajnaaBaaaleaacqaIWaamaeqaaOGaemOuai1aaSbaaSqaaiabd2gaTbqabaaakeaacqWGjbqsdaWgaaWcbaGaeGimaadabeaakiabdkfasnaaBaaaleaacqWGTbqBaeqaaOGaeyOeI0IaemOvay1aaSbaaSqaaiabdsha0jabdIgaObqabaaaaaGccaGLOaGaayzkaaGaeiOla4caaa@49B7@

When including the absolute refractory period, t_arp_, following each spike, the firing rate under injected constant current will be

r=1Tth+tarp=1τmln⁡(I0RmI0Rm−Vth)+tarp.
 MathType@MTEF@5@5@+=feaafiart1ev1aaatCvAUfKttLearuWrP9MDH5MBPbIqV92AaeXatLxBI9gBaebbnrfifHhDYfgasaacH8akY=wiFfYdH8Gipec8Eeeu0xXdbba9frFj0=OqFfea0dXdd9vqai=hGuQ8kuc9pgc9s8qqaq=dirpe0xb9q8qiLsFr0=vr0=vr0dc8meaabaqaciaacaGaaeqabaqabeGadaaakeaacqWGYbGCcqGH9aqpdaWcaaqaaiabigdaXaqaaiabdsfaunaaBaaaleaacqWG0baDcqWGObaAaeqaaOGaey4kaSIaemiDaq3aaSbaaSqaaiabdggaHjabdkhaYjabdchaWbqabaaaaOGaeyypa0ZaaSaaaeaacqaIXaqmaeaaiiGacqWFepaDdaWgaaWcbaGaemyBa0gabeaakiGbcYgaSjabc6gaUnaabmaabaWaaSaaaeaacqWGjbqsdaWgaaWcbaGaeGimaadabeaakiabdkfasnaaBaaaleaacqWGTbqBaeqaaaGcbaGaemysaK0aaSbaaSqaaiabicdaWaqabaGccqWGsbGudaWgaaWcbaGaemyBa0gabeaakiabgkHiTiabdAfawnaaBaaaleaacqWG0baDcqWGObaAaeqaaaaaaOGaayjkaiaawMcaaiabgUcaRiabdsha0naaBaaaleaacqWGHbqycqWGYbGCcqWGWbaCaeqaaaaakiabc6caUaaa@5B7E@

**Figure 3:**
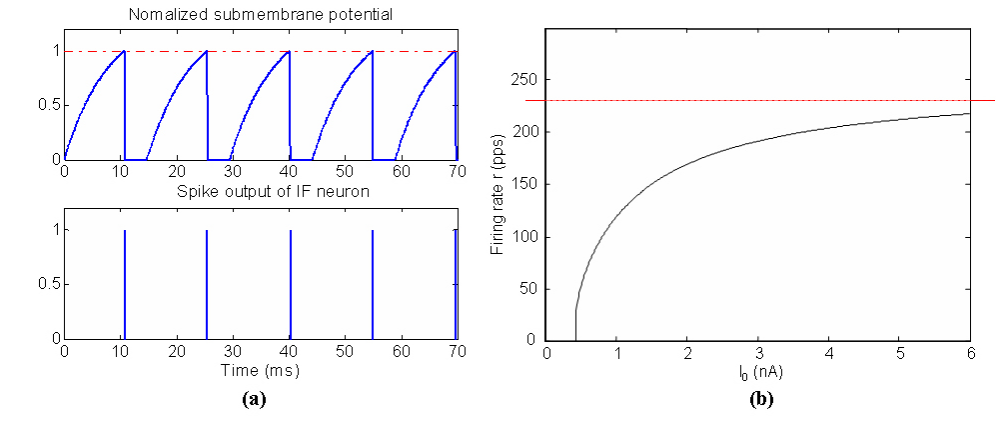
IF MN input-output relationships (a) Submembrane potential and spike output; (b) r-I relationship of the IF MN.

Figure 3 shows an example of the firing status and the input-output (I/O) relationship of the modelled MN, where the I/O function is described by the rate-intensity (r-I) relationship. The r-I curve gently bends over to level off at r_max _= 1/t_arp_.

**Figure 4:**
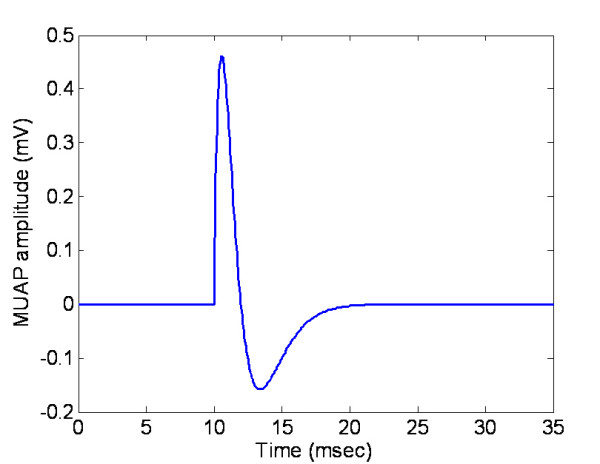
An example of MUAP waveform modelled by Eq.6.

The MUAP is another key factor in the ME channel. Generally it is the summation of action potentials generated by the simultaneously activated muscle fibers in the same motor unit. In this study, a mathematical model of the MUAP, which was proposed by Parker and Scott, was adopted for it agrees reasonably well with observed data [12]:

m(t)={a⋅p(t)=a⋅t(2−bt)exp⁡(−bt),0≤t0,otherwise,
 MathType@MTEF@5@5@+=feaafiart1ev1aaatCvAUfKttLearuWrP9MDH5MBPbIqV92AaeXatLxBI9gBaebbnrfifHhDYfgasaacH8akY=wiFfYdH8Gipec8Eeeu0xXdbba9frFj0=OqFfea0dXdd9vqai=hGuQ8kuc9pgc9s8qqaq=dirpe0xb9q8qiLsFr0=vr0=vr0dc8meaabaqaciaacaGaaeqabaqabeGadaaakeaacqWGTbqBcqGGOaakcqWG0baDcqGGPaqkcqGH9aqpdaGabaqaauaabaqaciaaaeaacqWGHbqycqGHflY1cqWGWbaCcqGGOaakcqWG0baDcqGGPaqkcqGH9aqpcqWGHbqycqGHflY1cqWG0baDcqGGOaakcqaIYaGmcqGHsislcqWGIbGycqWG0baDcqGGPaqkcyGGLbqzcqGG4baEcqGGWbaCcqGGOaakcqGHsislcqWGIbGycqWG0baDcqGGPaqkcqGGSaalaeaacqaIWaamcqGHKjYOcqWG0baDaeaacqaIWaamcqGGSaalaeaacqWGVbWBcqWG0baDcqWGObaAcqWGLbqzcqWGYbGCcqWG3bWDcqWGPbqAcqWGZbWCcqWGLbqzaaGaeiilaWcacaGL7baaaaa@6595@

where *a *is an amplitude modulator, *p(t) *determines the basic waveform of MUAP by the shape factor *b*, as shown in Fig. 4.

Substituting Eqs.5 and 6 into Eq.1, the SNR will be

SNR=163128b⋅[τmln⁡(RmI0RmI0−Vth)+tarp]−1,
 MathType@MTEF@5@5@+=feaafiart1ev1aaatCvAUfKttLearuWrP9MDH5MBPbIqV92AaeXatLxBI9gBaebbnrfifHhDYfgasaacH8akY=wiFfYdH8Gipec8Eeeu0xXdbba9frFj0=OqFfea0dXdd9vqai=hGuQ8kuc9pgc9s8qqaq=dirpe0xb9q8qiLsFr0=vr0=vr0dc8meaabaqaciaacaGaaeqabaqabeGadaaakeaacqWGtbWucqWGobGtcqWGsbGucqGH9aqpdaWcaaqaaiabigdaXaqaamaalaaabaGaeGOnayJaeG4mamdabaGaeGymaeJaeGOmaiJaeGioaGdaaiabdkgaIjabgwSixpaadmaabaacciGae8hXdq3aaSbaaSqaaiabd2gaTbqabaGccyGGSbaBcqGGUbGBdaqadaqaamaalaaabaGaemOuai1aaSbaaSqaaiabd2gaTbqabaGccqWGjbqsdaWgaaWcbaGaeGimaadabeaaaOqaaiabdkfasnaaBaaaleaacqWGTbqBaeqaaOGaemysaK0aaSbaaSqaaiabicdaWaqabaGccqGHsislcqWGwbGvdaWgaaWcbaGaemiDaqNaemiAaGgabeaaaaaakiaawIcacaGLPaaacqGHRaWkcqWG0baDdaWgaaWcbaGaemyyaeMaemOCaiNaemiCaahabeaaaOGaay5waiaaw2faaiabgkHiTiabigdaXaaacqGGSaalaaa@5CF1@

where *τ*_*m *_= *C*_*m*_*R*_*m *_is the membrane time constant; *I*_0 _is the constant current stimulus to MN, *V*_*th *_refers to the threshold voltage for MN firing, *t*_*arp *_represents the absolute refractory period, and *b *is the shape factor of MUAP. The detailed mathematical derivation procedure can be found in the Appendix.

It should be noted that the SNR defined by Eq.7 considers the noise as the amplitude variation only caused by the stochastic characteristics of the ME signal itself. In reality, there could be other noise sources, such as motion artifact, which could be arisen by movement of the muscles other than the target or the recording electrodes. Due to the main purpose, this study only focuses on the physiological factor effect on the SNR regardless any additional noise. Related analysis for the effect of the additional noise on ME control have been extensively investigated by Zhang [5]. Equation 7 clearly shows that the SNR of a single ME channel output is determined by the driving signal, I_0_, and the physiology of the motor unit.

### Simulation of the ME channel

In order to validate the mathematical derivation of Eq.7, simulations were performed. The values of physiological parameters were chosen based on previous experiments and modelling work [13,14]. Table 1 gives details of the physiological parameters in the model and simulation.

**Table 1:** 

**Physiological parameters**	**Value**
R_m _(MΩ)	25
C_m _(nF)	10
t_arp _(ms)	10
V_th _(mV)	16
I_0 _(nA)	6.5~16
b (s^-1^)	500~1500

Note: each parameter of IF MN is a lumped effect for the neuronal membrane is considered as a whole.

Simulation was carried out based on the ME signal generation process shown in Fig. 2. The SNR at the channel output, defined by Eq.1, was numerically calculated as the ratio of the mean and variance of the channel output y(t). Specifically,

E{y}=y¯=1n∑i=1nyi,
 MathType@MTEF@5@5@+=feaafiart1ev1aaatCvAUfKttLearuWrP9MDH5MBPbIqV92AaeXatLxBI9gBaebbnrfifHhDYfgasaacH8akY=wiFfYdH8Gipec8Eeeu0xXdbba9frFj0=OqFfea0dXdd9vqai=hGuQ8kuc9pgc9s8qqaq=dirpe0xb9q8qiLsFr0=vr0=vr0dc8meaabaqaciaacaGaaeqabaqabeGadaaakeaacqWGfbqrcqGG7bWEcqWG5bqEcqGG9bqFcqGH9aqpdaqdaaqaaiabdMha5baacqGH9aqpdaWcaaqaaiabigdaXaqaaiabd6gaUbaadaaeWbqaaiabdMha5naaBaaaleaacqWGPbqAaeqaaaqaaiabdMgaPjabg2da9iabigdaXaqaaiabd6gaUbqdcqGHris5aOGaeiilaWcaaa@4310@

and

Var{y}=1n−1∑i=1n(yi−y¯)2,
 MathType@MTEF@5@5@+=feaafiart1ev1aaatCvAUfKttLearuWrP9MDH5MBPbIqV92AaeXatLxBI9gBaebbnrfifHhDYfgasaacH8akY=wiFfYdH8Gipec8Eeeu0xXdbba9frFj0=OqFfea0dXdd9vqai=hGuQ8kuc9pgc9s8qqaq=dirpe0xb9q8qiLsFr0=vr0=vr0dc8meaabaqaciaacaGaaeqabaqabeGadaaakeaacqWGwbGvcqWGHbqycqWGYbGCcqGG7bWEcqWG5bqEcqGG9bqFcqGH9aqpdaWcaaqaaiabigdaXaqaaiabd6gaUjabgkHiTiabigdaXaaadaaeWbqaamaabmaabaGaemyEaK3aaSbaaSqaaiabdMgaPbqabaGccqGHsisldaqdaaqaaiabdMha5baaaiaawIcacaGLPaaadaahaaWcbeqaaiabikdaYaaaaeaacqWGPbqAcqGH9aqpcqaIXaqmaeaacqWGUbGBa0GaeyyeIuoakiabcYcaSaaa@4A60@

where n is the number of data points per MUAP train at an effective sampling rate of 10^4 ^samples per second.

## Results

**Figure 5:**
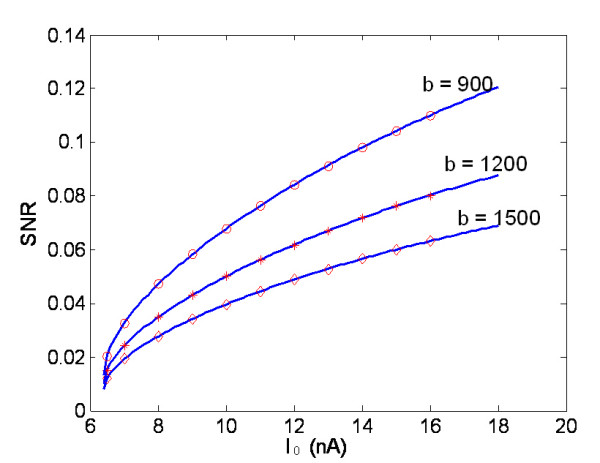
Relationship between SNR at ME channel output and effective driving current of MN (parameters are referred to Table 1; the solid lines are model results from Eq.7, and the symbolic lines are the simulation results).

Based on the model, it is possible to obtain the relationship between the neural control signal to the MU and the SNR at the ME channel output. Figure 5 shows such relationships for different MUs. It can be observed that the SNR increases with the intensity of the driving current, and the steepness of relationship curve is dependent on the shape factor of MUAP. It is well known that the driving current of the muscle is proportional to the voluntary contraction level. Therefore, the SNR of ME channel will be enhanced with an increasing contraction level.

**Figure 6:**
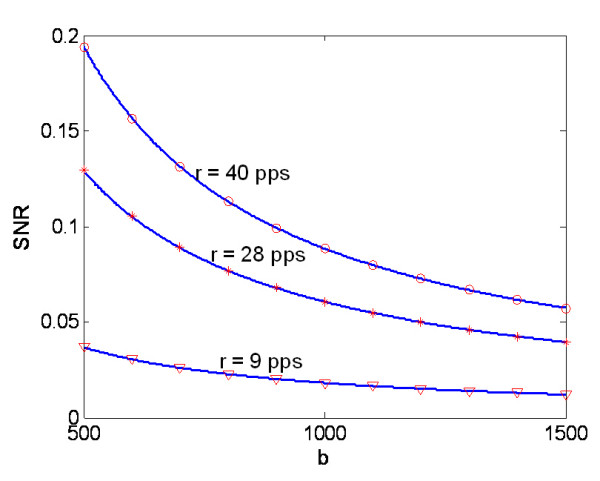
Theoretical and simulation results for SNR changes versus the shape factor, b, under different firing rates. (I_0 _= 6.5, 10 and 14.2 nA corresponding to the firing rate of 9, 28 40 pps respectively, and other parameters are referred to Table 1).

The model also can be used to investigate the effects of individual physiological characteristics on the SNR, which are difficult to obtain by experimental methods. According to Eq.7, the shape factor, which characterizes the distinction of MUAP, is a determinant of the SNR. Figure 6 shows that the SNR of the ME channel is inversely related to the shape factor *b *of the MUAP given an arbitrary firing rate. Implication of this result will be further discussed in the next session.

**Figure 7:**
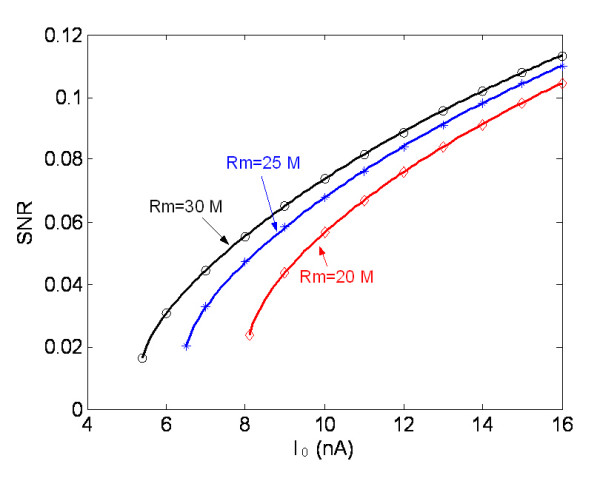
Effect of membrane resistance on the SNR at ME channel output (parameters are referred to Table 1; the solid lines are model results from Eq.7, and the symbolic lines are the simulation results).

Considering different types of motor units can be characterized by the membrane resistance of the MN [15,16], the relationship between membrane resistance of MN and the SNR at channel output was also studied. Figure 7 illustrates the SNR changes with the driving current intensity in different ME channels with different membrane resistance of MN.

**Figure 8:**
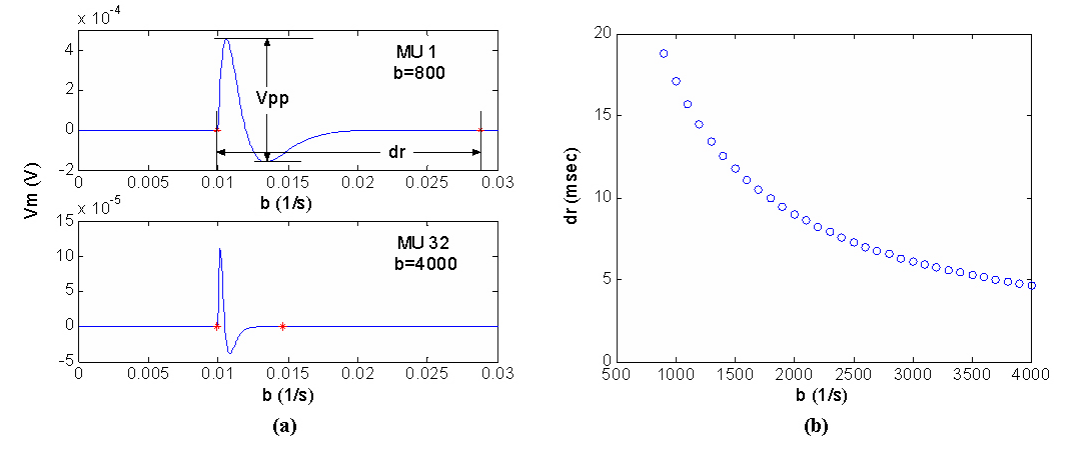
(a) examples of MUAP waveform with different *b *("*" indicates the deflection and return points for each MUAP; V_pp _refers to the peak-to-peak value and d_r _is the duration of MUAP); (b) The relationship between d_r _and *b*.

Each physiological parameter has its own dynamic range. Combining the current model with the existing experimental findings, it is possible to estimate the range of the SNR for a single ME channel during sustained contractions of human skeleton muscle. It was found that during the first four seconds of maximal effort, human limb muscle motor units may fire at 60–100 pps [17], while it is rare to record motor units firing more rapidly than 20 pps in normal limb muscles sustaining a contraction [18-20]. Some modelling work on motoneuron firing patterns suggested that the range of the firing rate of the motoneuron during a steady contraction is 8 to 50 pps [21]. On the other hand, the normal range for the MUAP duration is 5–20 ms. By choosing proper shape factor *b*, MUAP with specified duration can be synthesized by Eq.6. As shown in Fig. 8, for the MUAP duration ranging within 5–20 ms, b will be varied from 500 to 4000 s^-1^. Therefore, the maximum and minimum value for the SNR of a single ME channel can be estimated as

SNRmax⁡=λmax⁡63128bmin⁡−λmax⁡=5063128⋅600−50=0.2,
 MathType@MTEF@5@5@+=feaafiart1ev1aaatCvAUfKttLearuWrP9MDH5MBPbIqV92AaeXatLxBI9gBaebbnrfifHhDYfgasaacH8akY=wiFfYdH8Gipec8Eeeu0xXdbba9frFj0=OqFfea0dXdd9vqai=hGuQ8kuc9pgc9s8qqaq=dirpe0xb9q8qiLsFr0=vr0=vr0dc8meaabaqaciaacaGaaeqabaqabeGadaaakeaacqWGtbWucqWGobGtcqWGsbGudaWgaaWcbaGagiyBa0MaeiyyaeMaeiiEaGhabeaakiabg2da9maalaaabaacciGae83UdW2aaSbaaSqaaiGbc2gaTjabcggaHjabcIha4bqabaaakeaadaWcaaqaaiabiAda2iabiodaZaqaaiabigdaXiabikdaYiabiIda4aaacqWGIbGydaWgaaWcbaGagiyBa0MaeiyAaKMaeiOBa4gabeaakiabgkHiTiab=T7aSnaaBaaaleaacyGGTbqBcqGGHbqycqGG4baEaeqaaaaakiabg2da9maalaaabaGaeGynauJaeGimaadabaWaaSaaaeaacqaI2aGncqaIZaWmaeaacqaIXaqmcqaIYaGmcqaI4aaoaaGaeyyXICTaeGOnayJaeGimaaJaeGimaaJaeyOeI0IaeGynauJaeGimaadaaiabg2da9iabicdaWiabc6caUiabikdaYiabcYcaSaaa@61A8@

SNRmin⁡=λmin⁡63128bmax⁡−λmin⁡=863128⋅4000−8=0.004.
 MathType@MTEF@5@5@+=feaafiart1ev1aaatCvAUfKttLearuWrP9MDH5MBPbIqV92AaeXatLxBI9gBaebbnrfifHhDYfgasaacH8akY=wiFfYdH8Gipec8Eeeu0xXdbba9frFj0=OqFfea0dXdd9vqai=hGuQ8kuc9pgc9s8qqaq=dirpe0xb9q8qiLsFr0=vr0=vr0dc8meaabaqaciaacaGaaeqabaqabeGadaaakeaacqWGtbWucqWGobGtcqWGsbGudaWgaaWcbaGagiyBa0MaeiyAaKMaeiOBa4gabeaakiabg2da9maalaaabaacciGae83UdW2aaSbaaSqaaiGbc2gaTjabcMgaPjabc6gaUbqabaaakeaadaWcaaqaaiabiAda2iabiodaZaqaaiabigdaXiabikdaYiabiIda4aaacqWGIbGydaWgaaWcbaGagiyBa0MaeiyyaeMaeiiEaGhabeaakiabgkHiTiab=T7aSnaaBaaaleaacyGGTbqBcqGGPbqAcqGGUbGBaeqaaaaakiabg2da9maalaaabaGaeGioaGdabaWaaSaaaeaacqaI2aGncqaIZaWmaeaacqaIXaqmcqaIYaGmcqaI4aaoaaGaeyyXICTaeGinaqJaeGimaaJaeGimaaJaeGimaaJaeyOeI0IaeGioaGdaaiabg2da9iabicdaWiabc6caUiabicdaWiabicdaWiabisda0iabc6caUaaa@629E@

## Discussion

It is accepted that muscles generate force under two mechanisms, motor unit recruitment and firing rate modulation, both of which are determined by voluntary contraction level and neuromuscular physiology. In this paper, the SNR of a single ME channel was first modelled at the cellular level including the MN firing mechanisms. It provided a tool to understand the ME control process and to investigate influential factors individually, which would be very difficult to achieve by experimental methods.

### SNR sensitivity to the neural control signal

It is possible for the brain to judge the effort required and send suitable depolarizing signals to the MNs. Therefore, the stimulus intensity, which conveys the information of conscious contraction level, will determine the force generated by muscles. The recruitment of a motor unit depends on the neuronal firing threshold of its innervated MN. The one-to-one relationship between the occurrence of action potentials in a MN and in the muscle fibers it innervates infers that the CNS modulates the unit firing pattern by changing the input intensity of MN. When a larger force is required for the activated motor units, the firing rate will be increased. On one hand, the integral input of a MN can be equally modelled by an effective synaptic current [9,11,22], which is represented by a constant current, I_0_, in our model. On the other hand, indicated by Eqs.1 and 7, the SNR is largely sensitive to the mean firing rate of the motor unit among all the firing statistical characteristics. Therefore, the driving current of MN only influences the SNR at the ME channel output in terms of its mean value. Figure 5 clearly demonstrated that the SNR is enhanced with increased mean driving current.

### SNR sensitivity to MUAP morphology

Equation 7 shows that the SNR at the ME channel output is insensitive to the amplitude of the MUAP but inversely related to the shape factor *b*. The impact of the shape factor *b *on the morphology of the MUAP is studied by simulation. Thirty three MUAPs are synthesized with different shape factors based on Eq.6. Two examples are shown in Fig. 8(a). The durations of synthesized MUAPs are within the physiological range, normally 5~20 ms for human skeleton muscle [23]. It is observed that a larger *b *results in wider duration of the MUAP, as illustrated in Fig. 8.

**Figure 9:**
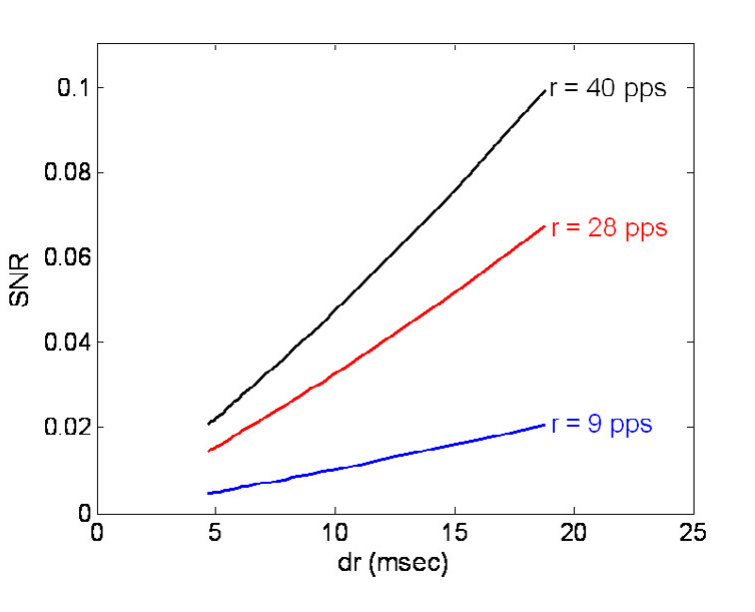
SNR changes against MUAP duration.

When the duration is defined as the interval from the first deflection from the baseline to the final return to the baseline [24], the relationship between the SNR and MUAP duration can be obtained, as shown in Fig. 9. Obviously, the SNR is proportional to the MUAP duration regardless of firing status. A similar conclusion was made in a previous study on single motor unit channel, the SNR is sensitive to a moment factor of MUAP [5], which is determined by the shape factor *b *as illustrated in the appendix. Physiologically, a MUAP is the temporal summation of the individual muscle fiber action potentials. The determining factors of MUAP duration are muscle fiber length, conduction velocity, and end-plate dispersion within the motor unit [25]. It is possible that poor SNR of ME channel is not caused by the ME control technique but resulted from the muscular physiology. Therefore, SNR should be treated differently according to the target muscle when it is used to evaluate the ME control performance.

### SNR related to the muscle contraction level

**Figure 10:**
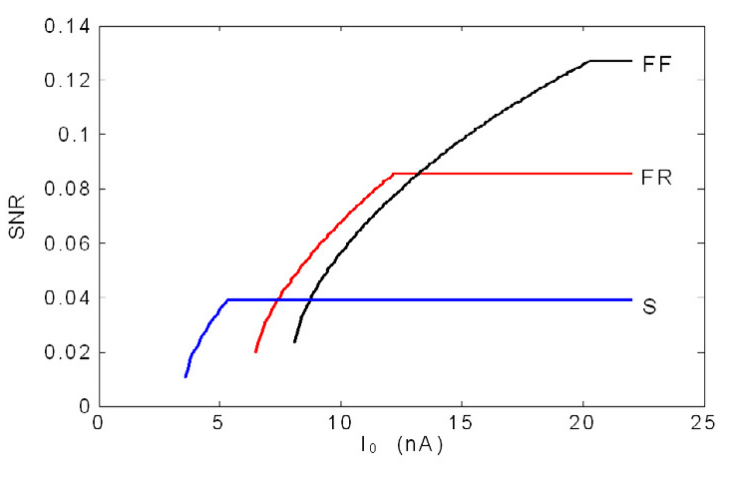
SNR changing against driving current in S, FR and FF types of motor units.

Strongly related to the muscle contraction level, the recruitment process is also important in determining the SNR of ME control. Motor units so far studied manifest considerable ranges of properties and can be categorized into three types based on their histochemical and mechanical properties as slow twitch (S), fast-twitch fatigue-resistant (FR) and fast-twitch fatigable (FF) [26]. During a muscle voluntary contraction, the motor units are recruited in an ascending order according to the size of their MNs [27], and generally recruited in order of type: S, FR, FF [26]. Different types of motor units have various firing thresholds and peak firing rates. With the increase of the muscle contraction level, the rates of low threshold units tend to saturate and higher-threshold units are recruited and discharge rates increase [28]. This physiological process will also result in variations in SNR at the ME channel output. In order to distinct the SNR characteristics in different types of motor unit channels, three ME channels were simulated by synthesizing S, FR and FF types of motor units. Simulation parameters were chosen according to previous studies [21], as shown in Table 2, while other parameters are the same as in Table 1. The result shown in Fig. 10 indicates that for an unsaturation state, smaller size motor units, which have higher membrane resistance and lower peak firing rate, would have higher SNR. However with the constraint of peak firing rate, a large size motor unit channel would have higher SNR at large stimulus intensity when the smaller size motor unit has already reached its peak firing rate. Obviously, there is an upper limit of SNR for specified a ME channel due to the firing rate saturation. According to the physiology of muscle contraction, increasing muscle contraction level will recruit the motor unit channels in an ascending order of SNR. In Zhang's study, the SNR measured on surface could reach 0.5. In comparison, the SNR of single ME control channel indicated by the Eq.10 is not high enough for accurate ME control. Other methods or technologies should be considered in order to enhance the ME control performance, such as ME control with multi channels. The limitation of the SNR in a single ME channel can be used as guidance for developing ME control techniques and training amputees to achieve optimal control.

**Table 2:** 

**Physiological parameters**	**S**	**FR**	**FF**
r_p _(pps) (peak firing rate)	16.7	35	50
R_m _(MΩ)	45	25	20
b (s^-1^)	1200	1200	1200

The modelling results indicate that large size motor units recruited at high contraction levels will enhance the SNR of the ME channels. Therefore, the SNR of a ME control channel is positively related to target force and will reach its peak value at the maximum contraction. A similar phenomenon was also reported in a previous experimental study [8].

According to above findings, ME control can be better understood and evaluated. For example, for small muscle with low contraction level task, SNR could be limited by the nature of the muscular physiologies, such as the driving current from the nerve, small size of the recruited motor units, etc. In the design of training strategies for amputee, muscles with large size of motor units should be chosen to achieve a high SNR of ME control.

## Conclusion

As an important measure of the ME control, the SNR of a single ME channel has been modelled including the physiological characteristics of MN and muscle unit. The effects of different physiological parameters on the SNR of the ME channel were investigated individually. The modelling results provided better a understanding of the relationship between the SNR of the ME channel and the neuromuscular physiology during a contraction. The major findings include:

1. The SNR of a single ME channel is highly related to the stimulus intensity of the motoneuron, which carries the information of the voluntary contraction level for a force task. As a result, it is clear that the performance of ME control would be enhanced with the increasing force task.

2. The SNR of a single ME channel is sensitive to the MUAP duration, which is mainly determined by the depolarization process, the muscle fiber length, conduction velocity, and end-plate dispersion within the motor unit. This conclusion may provide guidance to improve the performance of powered prostheses by considering the physiological factors in the control strategy design and the choice of proper target muscle for ME control.

3. The SNR of a single ME channel is generally ranged from 0.004 to 0.2. Techniques based on multi-channels are needed to improve the SNR for ME control.

4. Large size motor units will have higher SNR in the ME channel. Therefore, proper selection of the target muscle in a ME control may improve performance in terms of SNR.

## Appendix 1

In Zhang's model [8], the innervation process u(t) was regarded as stationary under the assumption that the muscle generates a constant force during isometric contraction. Therefore, u(t) was taken as a renewal point process. Following the single motor unit channel shown in Fig. 2, the output will be

*y*(*t*) = [*u*(*t*)*m(*t*)]^2 ^= *u*(*t*)*m^2^(*t*).

Following SNR definition of Eq.1,

E{y(t)}=r∫−∞∞m2(t)dt,
 MathType@MTEF@5@5@+=feaafiart1ev1aaatCvAUfKttLearuWrP9MDH5MBPbIqV92AaeXatLxBI9gBaebbnrfifHhDYfgasaacH8akY=wiFfYdH8Gipec8Eeeu0xXdbba9frFj0=OqFfea0dXdd9vqai=hGuQ8kuc9pgc9s8qqaq=dirpe0xb9q8qiLsFr0=vr0=vr0dc8meaabaqaciaacaGaaeqabaqabeGadaaakeaacqWGfbqrdaGadaqaaiabdMha5jabcIcaOiabdsha0jabcMcaPaGaay5Eaiaaw2haaiabg2da9iabdkhaYnaapedabaGaemyBa02aaWbaaSqabeaacqaIYaGmaaGccqGGOaakcqWG0baDcqGGPaqkcqWGKbazcqWG0baDaSqaaiabgkHiTiabg6HiLcqaaiabg6HiLcqdcqGHRiI8aOGaeiilaWcaaa@4654@

E{y2(t)}=r∫−∞∞m4(t)dt,
 MathType@MTEF@5@5@+=feaafiart1ev1aaatCvAUfKttLearuWrP9MDH5MBPbIqV92AaeXatLxBI9gBaebbnrfifHhDYfgasaacH8akY=wiFfYdH8Gipec8Eeeu0xXdbba9frFj0=OqFfea0dXdd9vqai=hGuQ8kuc9pgc9s8qqaq=dirpe0xb9q8qiLsFr0=vr0=vr0dc8meaabaqaciaacaGaaeqabaqabeGadaaakeaacqWGfbqrdaGadaqaaiabdMha5naaCaaaleqabaGaeGOmaidaaOGaeiikaGIaemiDaqNaeiykaKcacaGL7bGaayzFaaGaeyypa0JaemOCai3aa8qmaeaacqWGTbqBdaahaaWcbeqaaiabisda0aaakiabcIcaOiabdsha0jabcMcaPiabdsgaKjabdsha0bWcbaGaeyOeI0IaeyOhIukabaGaeyOhIukaniabgUIiYdGccqGGSaalaaa@4781@

and

Var{y(t)}=r{∫−∞∞m4(t)dt−λ(∫−∞∞m2(t)dt)2}.
 MathType@MTEF@5@5@+=feaafiart1ev1aaatCvAUfKttLearuWrP9MDH5MBPbIqV92AaeXatLxBI9gBaebbnrfifHhDYfgasaacH8akY=wiFfYdH8Gipec8Eeeu0xXdbba9frFj0=OqFfea0dXdd9vqai=hGuQ8kuc9pgc9s8qqaq=dirpe0xb9q8qiLsFr0=vr0=vr0dc8meaabaqaciaacaGaaeqabaqabeGadaaakeaacqWGwbGvcqWGHbqycqWGYbGCdaGadaqaaiabdMha5jabcIcaOiabdsha0jabcMcaPaGaay5Eaiaaw2haaiabg2da9iabdkhaYnaacmaabaWaa8qmaeaacqWGTbqBdaahaaWcbeqaaiabisda0aaakiabcIcaOiabdsha0jabcMcaPiabdsgaKjabdsha0bWcbaGaeyOeI0IaeyOhIukabaGaeyOhIukaniabgUIiYdGccqGHsisliiGacqWF7oaBdaqadaqaamaapedabaGaemyBa02aaWbaaSqabeaacqaIYaGmaaGccqGGOaakcqWG0baDcqGGPaqkcqWGKbazcqWG0baDaSqaaiabgkHiTiabg6HiLcqaaiabg6HiLcqdcqGHRiI8aaGccaGLOaGaayzkaaWaaWbaaSqabeaacqaIYaGmaaaakiaawUhacaGL9baacqGGUaGlaaa@5F34@

Finally we have

SNR=rk−r,
 MathType@MTEF@5@5@+=feaafiart1ev1aaatCvAUfKttLearuWrP9MDH5MBPbIqV92AaeXatLxBI9gBaebbnrfifHhDYfgasaacH8akY=wiFfYdH8Gipec8Eeeu0xXdbba9frFj0=OqFfea0dXdd9vqai=hGuQ8kuc9pgc9s8qqaq=dirpe0xb9q8qiLsFr0=vr0=vr0dc8meaabaqaciaacaGaaeqabaqabeGadaaakeaacqWGtbWucqWGobGtcqWGsbGucqGH9aqpdaWcaaqaaiabdkhaYbqaaiabdUgaRjabgkHiTiabdkhaYbaacqGGSaalaaa@3749@

where *r *is the mean firing rate of MN and

k=∫−∞∞m4(t)dt(∫−∞∞m2(t)dt)2.
 MathType@MTEF@5@5@+=feaafiart1ev1aaatCvAUfKttLearuWrP9MDH5MBPbIqV92AaeXatLxBI9gBaebbnrfifHhDYfgasaacH8akY=wiFfYdH8Gipec8Eeeu0xXdbba9frFj0=OqFfea0dXdd9vqai=hGuQ8kuc9pgc9s8qqaq=dirpe0xb9q8qiLsFr0=vr0=vr0dc8meaabaqaciaacaGaaeqabaqabeGadaaakeaacqWGRbWAcqGH9aqpdaWcaaqaamaapedabaGaemyBa02aaWbaaSqabeaacqaI0aanaaGccqGGOaakcqWG0baDcqGGPaqkcqWGKbazcqWG0baDaSqaaiabgkHiTiabg6HiLcqaaiabg6HiLcqdcqGHRiI8aaGcbaWaaeWaaeaadaWdXaqaaiabd2gaTnaaCaaaleqabaGaeGOmaidaaOGaeiikaGIaemiDaqNaeiykaKIaemizaqMaemiDaqhaleaacqGHsislcqGHEisPaeaacqGHEisPa0Gaey4kIipaaOGaayjkaiaawMcaamaaCaaaleqabaGaeGOmaidaaaaakiabc6caUaaa@4FA1@

Substituting MUAP function, Eq.6, into Eq.A6 yields

k=∫−∞∞m4(t)dt(∫−∞∞m2(t)dt)2=6320481b5(141b3)2=63128b.
 MathType@MTEF@5@5@+=feaafiart1ev1aaatCvAUfKttLearuWrP9MDH5MBPbIqV92AaeXatLxBI9gBaebbnrfifHhDYfgasaacH8akY=wiFfYdH8Gipec8Eeeu0xXdbba9frFj0=OqFfea0dXdd9vqai=hGuQ8kuc9pgc9s8qqaq=dirpe0xb9q8qiLsFr0=vr0=vr0dc8meaabaqaciaacaGaaeqabaqabeGadaaakeaacqWGRbWAcqGH9aqpdaWcaaqaamaapedabaGaemyBa02aaWbaaSqabeaacqaI0aanaaGccqGGOaakcqWG0baDcqGGPaqkcqWGKbazcqWG0baDaSqaaiabgkHiTiabg6HiLcqaaiabg6HiLcqdcqGHRiI8aaGcbaWaaeWaaeaadaWdXaqaaiabd2gaTnaaCaaaleqabaGaeGOmaidaaOGaeiikaGIaemiDaqNaeiykaKIaemizaqMaemiDaqhaleaacqGHsislcqGHEisPaeaacqGHEisPa0Gaey4kIipaaOGaayjkaiaawMcaamaaCaaaleqabaGaeGOmaidaaaaakiabg2da9maalaaabaWaaSaaaeaacqaI2aGncqaIZaWmaeaacqaIYaGmcqaIWaamcqaI0aancqaI4aaoaaWaaSaaaeaacqaIXaqmaeaacqWGIbGydaahaaWcbeqaaiabiwda1aaaaaaakeaadaqadaqaamaalaaabaGaeGymaedabaGaeGinaqdaamaalaaabaGaeGymaedabaGaemOyai2aaWbaaSqabeaacqaIZaWmaaaaaaGccaGLOaGaayzkaaWaaWbaaSqabeaacqaIYaGmaaaaaOGaeyypa0ZaaSaaaeaacqaI2aGncqaIZaWmaeaacqaIXaqmcqaIYaGmcqaI4aaoaaGaemOyaiMaeiOla4caaa@6956@

Thus, combined with Eqs.5 and A7, Eq.A5 for the SNR of ME control channel will be

SNR=163128b⋅[τmln⁡(RmI0RmI0−Vth)+tarp]−1.
 MathType@MTEF@5@5@+=feaafiart1ev1aaatCvAUfKttLearuWrP9MDH5MBPbIqV92AaeXatLxBI9gBaebbnrfifHhDYfgasaacH8akY=wiFfYdH8Gipec8Eeeu0xXdbba9frFj0=OqFfea0dXdd9vqai=hGuQ8kuc9pgc9s8qqaq=dirpe0xb9q8qiLsFr0=vr0=vr0dc8meaabaqaciaacaGaaeqabaqabeGadaaakeaacqWGtbWucqWGobGtcqWGsbGucqGH9aqpdaWcaaqaaiabigdaXaqaamaalaaabaGaeGOnayJaeG4mamdabaGaeGymaeJaeGOmaiJaeGioaGdaaiabdkgaIjabgwSixpaadmaabaacciGae8hXdq3aaSbaaSqaaiabd2gaTbqabaGccyGGSbaBcqGGUbGBdaqadaqaamaalaaabaGaemOuai1aaSbaaSqaaiabd2gaTbqabaGccqWGjbqsdaWgaaWcbaGaeGimaadabeaaaOqaaiabdkfasnaaBaaaleaacqWGTbqBaeqaaOGaemysaK0aaSbaaSqaaiabicdaWaqabaGccqGHsislcqWGwbGvdaWgaaWcbaGaemiDaqNaemiAaGgabeaaaaaakiaawIcacaGLPaaacqGHRaWkcqWG0baDdaWgaaWcbaGaemyyaeMaemOCaiNaemiCaahabeaaaOGaay5waiaaw2faaiabgkHiTiabigdaXaaacqGGUaGlaaa@5CF5@

## Abbreviations

b – shape factor of action potential

CNS – central nerve system

k – moment ratio

ME – myoelectric

MN – motoneuron

MSV – mean square value

MUAP – motor unit action potential

SNR – signal-to-noise ratio

r – mean firing rate

x(t) – myoelectric signal

y(t) – squared myoelectric signal

## Authors' contributions

HTM conceived of the study, proposed the model, and implemented the simulation. YTZ supervised the study and gave constructive advices to the research and the paper writing. Both authors read and approved the final manuscript.
